# Intranasal leptin improves survival after opioid overdose in a mouse model

**DOI:** 10.1186/s12967-021-02803-8

**Published:** 2021-03-31

**Authors:** Carla Freire, Huy Pho, Shannon Bevans-Fonti, Luiz U. Sennes, Vsevolod Y. Polotsky

**Affiliations:** 1 Johns Hopkins Sleep Disorders Center, 5501 Hopkins Bayview Circle, room 5A.50, Baltimore, MD 21224 USA; 2 Otolaryngology Department, University of São Paulo, Sao Paulo, Brazil

**Keywords:** Opioid reversal agents, Leptin, Blood brain barrier, Opioid overdose

## To the editor:

It is critical to develop drugs that prevent deaths induced by opioids. More than 120 daily deaths in the United States are attributed to opioid overdose and mortality is accelerating with the intersection between the opioid crisis and the COVID-19 pandemic [1]. Naloxone is the only available drug capable of reversing opioid’s adverse effects and preventing death. However, naloxone has a limited use because it reverses analgesia and induces withdrawal [2]. We have previously reported that intranasal (IN) leptin prevents opioid induced respiratory depression, the main cause of death related to opioids [3]. In this translational study, we investigate if the intranasal route effectively delivers leptin to the brain, where it acts on respiratory control centers [4–6], and if it prevents opioid-related deaths.

In total, 61 male, 12 weeks old, C57BL/6 J mice were used in the study. Food and water were provided ad libitum. Mice were housed in a standard laboratory environment at 24–26 °C in the 12-h light/dark cycle (9 am–9 pm lights-on). The study was approved by the Johns Hopkins University Animal Use and Care Committee and complied with the American Physiological Society Guidelines. To determine leptin delivery to the brain, 10 mice were randomized to receive IN leptin at 1.2 mg/kg in 1% BSA or IN vehicle (1% BSA). IN administration was performed under Isoflurane anesthesia with the mouse in supine position as previously described [3]. Mice were euthanized 20 min after the injection, brains were harvested and olfactory bulbs, medullas and hypotalami were isolated, quick frozen and stored at − 80 °C. For leptin level measurements brain tissue was homogenized in 160 mM KCL, 25 mM HEPES, 0.2% Triton X-100 and protease inhibitors, protein concentrations were determined using a BioRad DC Protein kit and ELISA was performed with a Millipore kit. To determine survival probability, mice received IN leptin at 1.2 mg/kg (n = 26) or IN vehicle (n = 25) followed by intraperitoneal (IP) injection of morphine at 400 mg/kg 30 min after the IN treatment. Mice were observed for 24 h in video monitored cages and survival time after morphine injection was recorded. Mice that survived after the observation period were euthanized. Continuous variables are presented as means ± SEMs and categorical variables are represented as proportions. We performed comparisons between leptin concentrations after IN leptin or IN vehicle by independent samples t-test. Survival probabilities were analyzed by the Kaplan–Meier method and compared using the log-rank test. Statistical analyses were performed using R Statistical Software (version 4.0.2; R Foundation for Statistical Computing, Vienna, Austria) and p < 0.05 was considered significant.

**Fig. 1:**
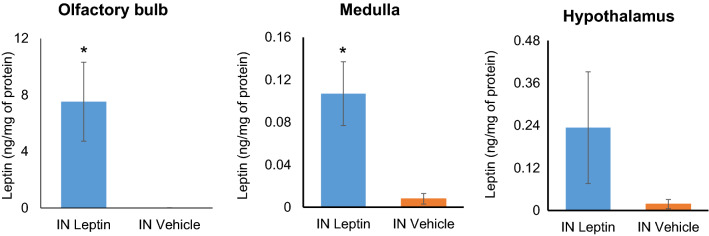
Leptin levels measured in the olfactory bulb, medulla and hypothalamus 20 min after administration of leptin or vehicle. Leptin levels were higher in mice that received IN leptin (n = 5) when compared to mice that received IN vehicle (n = 5) in the olfactory bulb and medulla. *p < 0.05. IN, intranasal

The IN route delivered leptin to the brain in mice (Fig. 1). Comparison between leptin levels 20 min after IN administration of leptin or vehicle showed a significant increase in the olfactory bulb (7.529 ± 2.84 ng/mL vs 0 ± 0.004 ng/mL, p = 0.05) and medulla (0.107 ± 0.03 ng/mL vs 0.008 ± 0.005 ng/mL, p = 0.03). We observed a trend for increase in the hypothalamus (0.234 ± 0.151 ng/mL vs 0.018 ± 0.013 ng/mL, p = 0.18).

**Fig. 2:**
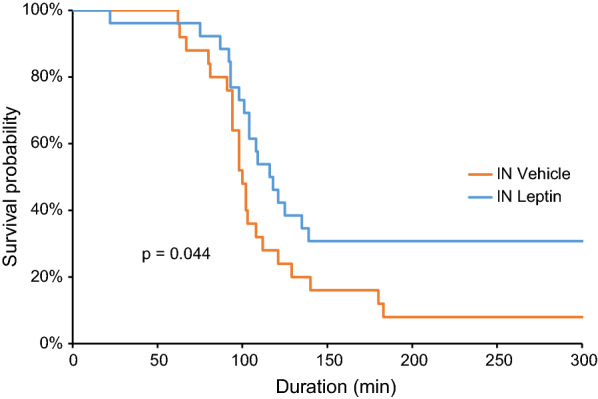
Kaplan–Meier curves comparing survival between mice that received IN vehicle and mice that received IN leptin. IN leptin administered 30 min prior to morphine (400 mg/kg) significantly improved survival probability. p = 0.044. IN, intranasal

IN leptin improved the survival probability after a 400 mg/kg dose of morphine IP (p = 0.044). Mice that received IN vehicle had a 92% mortality rate with a median survival time of 100 min, whereas mice that received IN leptin had a 69% mortality rate with a median survival time of 117 min. Kaplan–Meier curves are shown in Fig. 2, mice were monitored for 24 h but no deaths occurred after 183 min.

The main novel finding of our study is that IN leptin reduced mortality associated with morphine overdose in mice. Several mechanisms may contribute to this 25% reduction in mortality. Data from previous studies showing that leptin mitigates the effects of opioids by improving upper airway patency and control of breathing suggest that decrease in mortality is mainly due to attenuation of respiratory failure [3]. However, Bubier et al. did not observe a correlation between respiratory depression, recovery time and survival time in different mouse strains [7]. Thus, the adverse effects of morphine on other systems may play a role in morphine induced mortality.

Leptin is produced by the adipose tissue and acts in the central nervous system to regulate metabolic homeostasis, fertility, immune function and breathing, among others [8]. Investigators have shown that the respiratory effects of leptin occur in medullary and hypothalamic centers in the brain [4–6, 9] and in the carotid body [10]. Leptin is currently approved for the treatment of patients with lipodystrophy and leptin deficiency. One of the limitations for the advance of leptin as a therapeutic agent is leptin resistance with numerous factors that contribute to leptin transport to the brain [8]. Leptin does not diminish opioid analgesia [3], and should not induce opioid withdrawal. Therefore, our finding that the IN route effectively delivers leptin to the brain has translational significance.

This study has limitations, including: (a) the lack of monitoring of opioid side effects such as respiratory depression and cardiac function; (b) the use of anesthesia for IN administration of leptin may have had an impact on mortality, (c) the anatomical differences between mouse and human olfactory systems might affect the translation of this therapy. Finally, the effects of leptin in females or after chronic opioid administration were not tested.

In conclusion, our results show that the IN route is effective for the delivery of leptin to the brain in mice and that IN leptin attenuates morphine-induced mortality. Our data suggest that IN leptin may be considered for prevention of opioid overdose, especially when analgesic properties of opioids have to be preserved.

## Data Availability

The datasets used and/or analysed during the current study are available from the corresponding author on reasonable request.

## Abbreviations

IN: Intranasal
IP: Intraperitoneal
